# Perinatal Resveratrol Therapy Prevents Hypertension Programmed by Maternal Chronic Kidney Disease in Adult Male Offspring: Implications of the Gut Microbiome and Their Metabolites

**DOI:** 10.3390/biomedicines8120567

**Published:** 2020-12-04

**Authors:** Chien-Ning Hsu, Chih-Yao Hou, Guo-Ping Chang-Chien, Sufan Lin, Hung-Wei Yang, You-Lin Tain

**Affiliations:** 1 Department of Pharmacy, Kaohsiung Chang Gung Memorial Hospital, Kaohsiung 833, Taiwan; cnhsu@cgmh.org.tw; 2 School of Pharmacy, Kaohsiung Medical University, Kaohsiung 807, Taiwan; 3 Department of Seafood Science, National Kaohsiung University of Science and Technology, Kaohsiung 811, Taiwan; chihyaohou@gmail.com; 4 Center for Environmental Toxin and Emerging-Contaminant Research, Cheng Shiu University, Kaohsiung 833, Taiwan; guoping@csu.edu.tw (G.-P.C.-C.); linsufan2003@gmail.com (S.L.); 5 Super Micro Mass Research and Technology Center, Cheng Shiu University, Kaohsiung 833, Taiwan; 6 Institute of Medical Science and Technology, National Sun Yat-Sen University, Kaohsiung 804, Taiwan; 7 Department of Pediatrics, Kaohsiung Chang Gung Memorial Hospital and Chang Gung University College of Medicine, Kaohsiung 833, Taiwan; 8 Institute for Translational Research in Biomedicine, Kaohsiung Chang Gung Memorial Hospital and Chang Gung University College of Medicine, Kaohsiung 833, Taiwan

**Keywords:** asymmetric dimethylarginine, developmental origins of health and disease (DOHaD), hypertension, nitric oxide, oxidative stress, chronic kidney disease, resveratrol, gut microbiota, short chain fatty acid, trimethylamine-*N*-oxide

## Abstract

The gut microbiota plays a critical role in kidney disease and hypertension; however, whether maternal chronic kidney disease (CKD)-induced offspring hypertension is associated with alterations of the microbiota and microbial metabolites remains elusive. Using rat as an animal model, we conducted a maternal adenine-induced CKD model to examine whether adult male offspring develop hypertension and kidney disease. As resveratrol has antioxidant and prebiotic properties, we also aimed to elucidate whether its use in pregnancy and lactation can benefit hypertension programmed by maternal CKD via mediation of the gut microbiota and oxidative stress. Female Sprague-Dawley rats received regular chow (C) or chow supplemented with 0.5% adenine (CKD) from 3 weeks before pregnancy until lactation. One group of the adenine-induced CKD pregnant rats received resveratrol (R; 50 mg/L) in drinking water during gestation and lactation. Male offspring were divided into three groups: C, CKD, and CKD+R. The microbial metabolites analyzed were short chain fatty acids (SCFAs) in feces and trimethylamine (TMA)/trimethylamine N-oxide (TMAO) in plasma. We found perinatal resveratrol therapy protected against maternal CKD-induced hypertension in adult male offspring. The overall microbial compositions and diversity of bacterial community in the three groups were different. Resveratrol therapy increased α-diversity, decreased the *Firmicutes* to *Bacteroidetes* ratio, and increased the abundance of the genera *Lactobacillus* and *Bifidobacterium*. Perinatal resveratrol therapy increased plasma TMA levels but decreased the plasma TMAO-to-TMA ratio. Although resveratrol had negligible effect on fecal concentrations of SCFAs, it increased G-protein coupled receptor-41 (GPR41) protein levels in the offspring’s kidneys. Additionally, resveratrol therapy increased plasma levels of L-arginine and the L-arginine-to-ADMA ratio (AAR), and decreased oxidative stress. Overall, the protective effects of resveratrol against programmed hypertension are related to gut microbiome remodeling, including an increased abundance of beneficial microbes, mediation of the TMA-TMAO pathway, and alterations of SCFA receptors. Our results highlighted that targeting the microbiome and their metabolites might be potential therapeutic strategies to prevent maternal CKD-induced adverse pregnancy and offspring outcomes.

## 1. Introduction

Chronic kidney disease (CKD) is a highly prevalent disease that affects nearly 10% of the world’s population [1]. At least 3–4% of women of reproductive age are complicated by this condition [2]. Pregnant women with CKD are at risk of adverse maternal and perinatal outcomes [3]. However, the existing literature is sparse regarding the long-term effects of maternal CKD on renal outcomes in adult offspring. We previously reported that maternal CKD increases the risks of developing hypertension and renal hypertrophy in adult male rat offspring [4]. Current evidence suggests that hypertension and CKD are interconnected, and both can originate in early life [5,6,7]. This concept, namely “developmental origins of health and disease (DOHaD)”, has been used widely to demonstrate that the developing fetus exposed to a suboptimal in utero environment increases the risk for developing many chronic diseases in adulthood [8].

Recently, the interconnection between early-life gut microbiota dysbiosis and later risk of adult diseases has been shown [9]. Several mechanisms have been shown to link gut microbiota dysbiosis to hypertension [10,11], including alterations of microbial composition and metabolites, mediation of short chain fatty acids (SCFAs) and their receptors, increased production of trimethylamine-N-oxide (TMAO), an impaired nitric oxide (NO) pathway, and increased oxidative stress. Data from experimental and clinical studies of CKD support the concept that dysbiotic gut microbiota, increased trimethylamine (TMA) and TMAO, reduced SCFAs, and increased asymmetric and symmetric dimethylarginine (ADMA and SDMA, endogenous inhibitors of NO synthase) collectively deteriorate the progression of CKD and hypertension [10,11,12,13]. TMAO, TMA, and SCFAs are gut microbiota-derived metabolites. Flavin monooxygenase (FMO) isoforms catalyze the formation of TMAO from gut microbiota-derived TMA. Both TMA and TMAO are known uremic toxins implicated in cardiovascular risk [13,14,15]. Recent research suggests that SCFAs have a positive effect on hypertension via mediation of SCFA receptors and anti-inflammation, or the other way [10,16]. Our previous studies demonstrated that early interventions targeting TMAO and SCFAs can protect adult offspring against hypertension programmed by maternal a high-fructose or high-fat diet [17,18]. It was also reported that an impaired ADMA-NO pathway seems to be associated with hypertension of developmental origin [19,20]. Thus, we first aimed to examine whether maternal adenine-induced CKD can induce hypertension and kidney damage in adult offspring related to changes in gut microbiota composition, alterations of microbe-derived metabolite TMA and SCFAs, and an impaired NO pathway.

With growing knowledge of the developmental programming, early-life interventions to halt or reverse the programming processes by so-called reprogramming [21] offers a novel strategy to prevent the development of hypertension and kidney disease [6,7]. Resveratrol, a natural polyphenol with antioxidant properties, has a wide range of health benefits [22,23]. Resveratrol has been used as a reprogramming intervention to prevent hypertension in several models of developmental programming [24,25,26,27]. Emerging data suggest its therapeutic potential may be because resveratrol can alter the gut microbiota and act like a prebiotic [28]. Given the close association among resveratrol, gut microbiota, and programmed hypertension, the second aim of this study was to verify whether maternal resveratrol therapy can prevent maternal CKD-induced hypertension and whether the beneficial effects of resveratrol are related to modulation of gut microbiota composition and metabolites.

## 2. Experimental Section

### 2.1. Animals and Study Design

**Figure 1:**
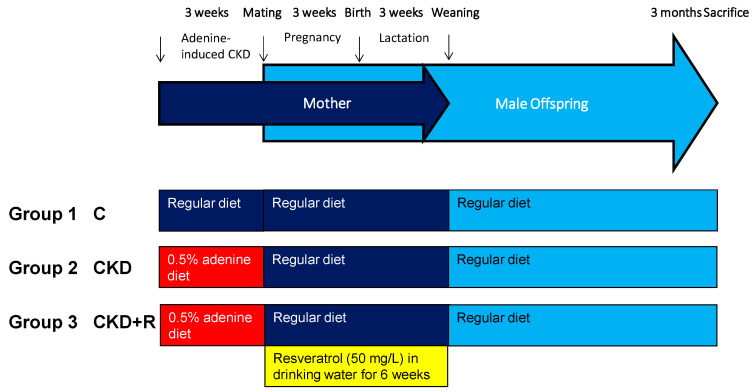
Schematic illustration of study design to establish a maternal adenine-induced chronic kidney disease (CKD) and/or resveratrol (R)-treated rat model to evaluate the protective effects of perinatal resveratrol therapy in male offspring at 3 months of age. The arrows indicate the different stages of development.

Virgin Sprague-Dawley (SD) rats were purchased at BioLASCO Taiwan Co., Ltd. (Taipei, Taiwan) for mating. Rats were housed in our animal center accredited by the Association for Assessment and Accreditation of Laboratory Animal Care International. As described previously [4], female SD rats aged 8 weeks received a regular diet (C group) or a diet supplemented with 0.5% adenine (CKD group) for 3 weeks. At 11 weeks of age, female rats were caged with male rats until mating. The presence of a copulatory plug in the vaginal-cervical region confirmed mating. One group (CKD+R) of the adenine-treated pregnant rats received resveratrol (50 mg/L; Sigma-Aldrich, St. Louis, MO, USA) in drinking water during gestation and lactation (i.e., a total of 6 weeks) to cover the period of nephrogenesis. The doses of resveratrol used here are based on our earlier reports [25,26,27]. Since males are more susceptible to develop hypertension than females [29], only male offspring was selected and used in subsequent experiments. After birth, the size of the litter was standardized to eight individuals. Male offspring were assigned to three experimental groups (*n* = 8 per group): C, CKD, and CKD+R. The offspring were weaned at 3 weeks of age and put onto regular chow. The experimental protocol is illustrated in Figure 1.

We used an indirect tail-cuff method (CODA monitor, Kent Scientific Corporation, Torrington, CT, USA) to measure blood pressure (BP) in conscious rats every 2 weeks, as described previously [4]. All offspring were sacrificed at 12 weeks of age. Heparinized blood samples were collected at the end of the study. The kidneys were subsequently collected. The kidneys were harvested and stored at −80 °C in a freezer for further analysis. Animal care and experiments were conducted following established guidelines for the Care and Use of Laboratory Animals of the National Institutes of Health. All animal studies were approved by the Institutional Animal Ethics Committee (IACUC) of Chang Gung Memorial Hospital (Permit Number 2019081501, 16 March 2020).

### 2.2. Liquid Chromatography-Mass Spectrometry Analysis

According to previously described methods [17], plasma levels of TMA, TMAO, and dimethylamine (DMA, the metabolite of TMA and TMAO) were determined by LC-MS. For the analysis, an Agilent 6410 Series Triple Quadrupole mass spectrometer (Agilent Technologies, Wilmington, DE, USA) with an electrospray ionization source was used. The Agilent Technologies 1200 HPLC system was equipped with an autosampler and a binary pump. We used diethylamine as an internal standard. Chromatographic separation was performed on a SeQuant ZIC-HILIC column (150 × 2.1 mm, 5 μm; Merck KGaA, Darmstadt, Germany) protected by an Ascentis C18 column (2 cm × 4 mm, 5 μm; Merck KGaA). TMA, TMAO, and DMA were monitored in multiple-reaction-monitoring mode using characteristic precursor-product ion transitions: *m*/*z* 60.1→44.1, *m*/*z* 76.1→58.1, and *m*/*z* 46.1→30, respectively.

### 2.3. Gas Chromatography-Mass Spectrometry

Gas chromatography-mass spectrometry (Agilent Technologies 7890B, Wilmington, DE, USA) with an automated sampler was used to measure fecal SCFA concentrations, as described previously [17]. Chromatographic separation was achieved using a DB-FFAP column (30 m × 0.25 mm, 0.25 µm; Agilent Technologies, Wilmington, DE, USA). Injection was performed at 240 °C; the injection volume was 1 μL with a split ratio of 5:1. We used 2-ethylbutiric acid as an internal standard. The mass spectrometry (MS) was operated using an ionization voltage of 70 eV, an ion source temperature of 230 °C, and a quadrupole temperature of 150 °C. Six SCFAs (acetate, propionate, isobutyrate, butyrate, isovalerate, and valerate) in the gas-phase ions were detected according to their mass-to-charge (*m*/*z*) ratio.

### 2.4. High-Performance Liquid Chromatography 

Plasma L-arginine (the substrate for NO synthase), L-citrulline (the precursor of L-arginine), ADMA, and SDMA levels were measured using HPLC (HP series 1100, Agilent Technologies, Inc., Santa Clara, CA, USA) with a derivatization reagent, ortho-phthaldialdehyde-3-mercaptopropionic acid (OPA-3-MPA) [12].

### 2.5. Immunohistochemistry Staining of 8-Hydroxydeoxyguanosine

According to previously described methods [26], paraffin-embedded tissues sectioned at 3 μm thickness were deparaffinized in xylene and rehydrated in a graded series of ethanol and washed in phosphate-buffered saline (PBS). 8-Hydroxydeoxyguanosine (8-OHdG) is a DNA oxidation product that was measured to assess DNA damage. Following blocking with immunoblock (Thermo Fisher Scientific Inc., Waltham, MA, USA), the sections were incubated for 1 h at room temperature with an anti-8-OHdG antibody (1:500, GeneTex Inc., Irvine, CA, USA). Quantitative analysis of number of 8-OHdG-positive cells per field (X400) was performed in the renal sections [26].

### 2.6. Western Blot

We analyzed three SCFA receptors, including G-protein coupled receptor-41 (GPR41), -43 (GPR43), and -91 (GPR91). Kidney samples were subjected to electrophoresis, Western blot, and antibody incubation, as described previously [17,18]. Briefly, 200 μg of kidney cortex was loaded on a 10% polyacrylamide gel and separated by electrophoresis (200 volts, 90 min). The proteins were then electrotransferred to a nitrocellulose membrane (GE Healthcare Bio-Sciences Corp., Piscataway, NJ, USA). The membranes were incubated with Ponceau S red (PonS) stain solution (Sigma-Aldrich, St. Louis, MO, USA) for 10 min on the rocker. After blocking with PBS-Tween (PBS-T) containing 5% dry milk, the blot was incubated in a dilute solution of primary antibody. We used the following primary antibodies: a rabbit polyclonal anti-rat GPR41 antibody (1:500, overnight incubation; USBiological, Salem, MA, USA), a rabbit polyclonal anti-rat GPR43 antibody (1:500, overnight incubation; Millipore, Burlington, MA, USA), and a rabbit polyclonal anti-rat GPR91 antibody (1:1000, 1.5 h incubation; Novus Biologicals, Littleton, CO, USA). Bound antibodies were detected using a secondary antibody. Later, the membrane was imaged using an enhanced chemiluminescence reagent (PerkinElmer, Waltham, MA, USA) and analyzed using Quantity One Analysis software (Bio-Rad, Hercules, LA, USA). Band density was calculated as the integrated optical density (IOD) minus the background value, normalized to PonS staining to correct the variations in total protein loading. The protein abundance was represented as IOD/PonS.

### 2.7. Metagenomics Analysis of the Gut Microbiota

Frozen fecal samples were analyzed with metagenomics using the methods published previously [30]. The amplicons were sequenced on an Illumina MiSeq sequencer (Illumina, CA, USA) at Biotools Co., Ltd. (Taipei, Taiwan). The sequences were processed using QIIME version 1.9.1. Sequences were clustered into operational taxonomic units (OTUs) using a threshold of 97% by USEARCH algorithm. The phylogenetic relationships were determined based on a representative sequence alignment using Fast-Tree. We compared patterns of α- and β-diversity for microbial communities [31]. We measured α-diversity as the observed richness and evenness of the taxa using Shannon’s index. We evaluated the β-diversity changes in gut microbiota across groups by analysis of similarities (ANOSIM) and partial least squares discriminant analysis (PLS-DA). We used the linear discriminant analysis effect size (LEfSe) to detect taxa with differential abundance among groups. The threshold of the linear discriminant was set to 4.

### 2.8. Statistical Analysis

Data were analyzed by the Statistical Package for the Social Sciences software 15.0 (SPSS Inc., Chicago, IL, USA) and expressed as means ± the standard error of the mean (SEM). Comparisons within the three groups were analysis by one-way analysis of variance (ANOVA), followed by Tukey’s post hoc test. Systolic BP (SBP) was analyzed by two-way repeated-measures analysis of variance and Tukey’s post hoc test. Significance was determined at a probability value of <0.05.

## 3. Results

### 3.1. Blood Pressure and Renal Function

**Figure 2:**
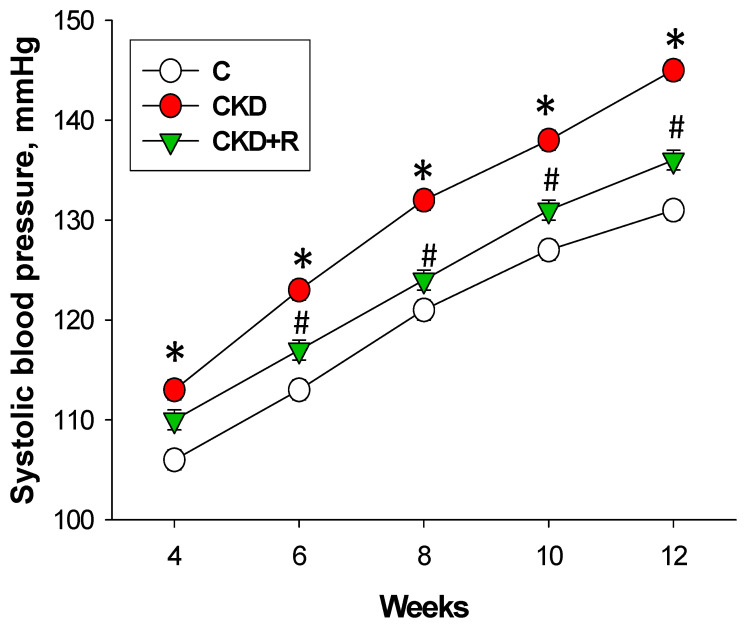
Effect of maternal CKD and R treatment on systolic blood pressure in 12-week-old male offspring. *N* = 7–8/group; * *p* < 0.05 vs. C; # *p* < 0.05 vs. CKD.

One pup was dead after birth in the CKD+R group. The body weight (BW) was higher in the CKD+R group than the C and CKD group (Table 1). Maternal CKD caused a higher kidney weight and kidney weight-to-BW ratio in the CKD and CKD+R group. Longitudinal measurement of systolic BP (SBP) from 4 to 12 weeks of age showed that maternal CKD caused increases in SBP (Figure 2), while the elevation of SBP was prevented by maternal resveratrol therapy from 6 to 12 weeks of age. At 12 weeks of age, diastolic BP and mean arterial pressure were also elevated in the CKD group compared with the controls. As shown in Table 1, CKD and resveratrol had negligible effect on renal function as determined by the blood creatinine level.

### 3.2. Gut Microbiota Composition

**Figure 3:**
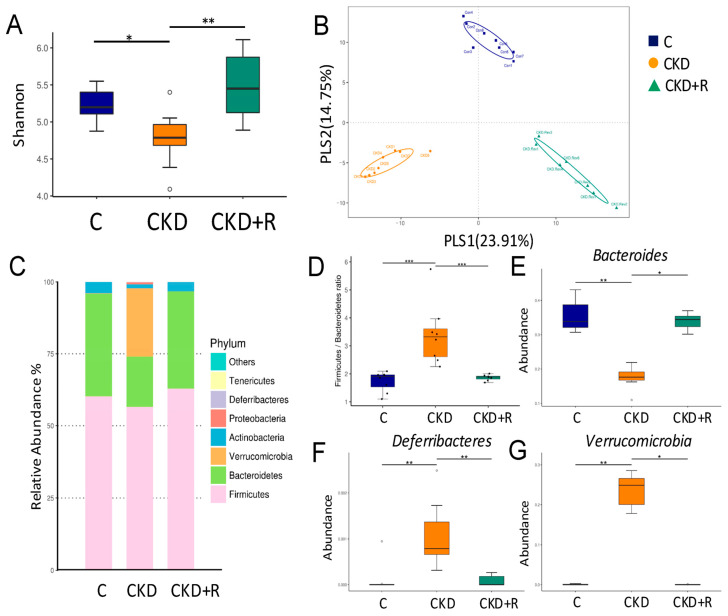
Effects of maternal CKD and R treatment on the gut microbiota in 12-week-old male offspring. (**A**) α-diversity represented by Shannon’s diversity indexes. (**B**) β-diversity changes in gut microbiota across groups by partial least squares discriminant analysis (PLS-DA). (**C**) Relative abundance of top 10 phyla in the three groups. (**D**) The *Firmicutes* to *Bacteroidetes* ratio. Relative abundance of the phyla (**E**) *Bacteroidetes*, (**F**) *Deferribacteres*, and (**G**) *Verrucomicrobia*. *N* = 7–8/group; * *p* < 0.05; ** *p* < 0.01; *** *p* < 0.001.

We next investigated changes in the gut microbiota. We first examined α-diversity using the Shannon diversity index to determine species richness and relative abundance [30]. Figure 3A shows that the CKD group exhibited a loss of α-diversity compared with the controls (*p* = 0.038), while perinatal resveratrol treatment significantly expanded α-diversity in the CKD+R group compared with the CKD group (*p* = 0.007). We next executed two different β-diversity analysis techniques to compare the bacterial community similarity using partial least squares discriminant analysis (PLS-DA) and analysis of similarities (ANOSIM). As shown in Figure 3B, the scatterplots of PLS-DA analysis were cleanly separated. ANOSIM confirmed a significant variation in the gut microbiota among the three groups (all *p* < 0.05), indicating that the three groups had distinct enterotypes. The relative abundance of the dominant bacteria phyla is shown in Figure 3C, including *Firmicutes*, *Bacteroidetes, Verrucomicrobia, Actinobacteria, Proteobacteria,* and *Deferribacteres*. The *Firmicutes* to *Bacteroidetes* (F/B) ratio has been considered a signature for hypertension [11]. We observed that maternal CKD significantly increased SBP and this ratio concurrently, which was restored by maternal resveratrol treatment (Figure 3D). Additionally, the CKD group had a lower abundance of phylum *Bacteroides* than the controls (Figure 3E), while this reduction was restored by the resveratrol treatment in the CKD+R group. The abundance from the phyla *Deferribacteres* and *Verrucomicrobia* was expanded by maternal CKD, while resveratrol treatment prevented this (Figure 3F,G).

**Figure 4:**
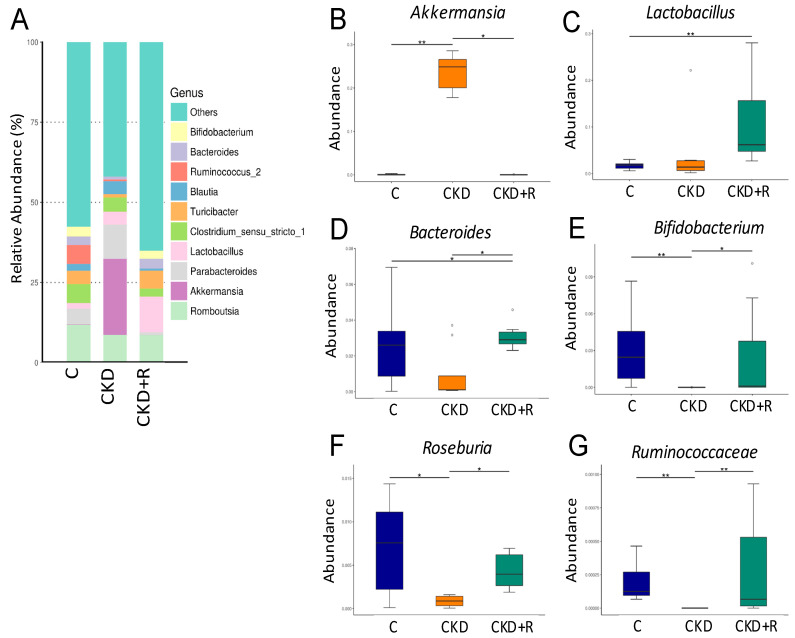
Effects of maternal CKD and R treatment on the gut microbiota in 12-week-old male offspring. (**A**) Relative abundance of the top 10 genera in the three groups. Abundance of the genera (**B**) *Akkermansia*, (**C**) *Lactobacillus*, (**D**) *Bacteroides*, (**E**) *Bifidobacterium*, (**F**) *Roseburia*, and (**G**) *Ruminococcaceae* in the three groups. * *p* < 0.05; ** *p* < 0.01.

Figure 4A shows the relative abundance of major genera in the three groups. At the genus level, CKD significantly increased genus *Akkermansia* abundance compared with the controls, which was restored by resveratrol (Figure 4B). The CKD group had a higher abundance of the genus *Lactobacillus* compared with the other two groups (Figure 4C). Additionally, several genera, including *Bacteroides, Bifidobacterium, Roseburia,* and *Ruminococcaceae*, were lower in the CKD group vs. controls (Figure 4D–G). These decreases were restored by perinatal resveratrol therapy.

**Figure 5:**
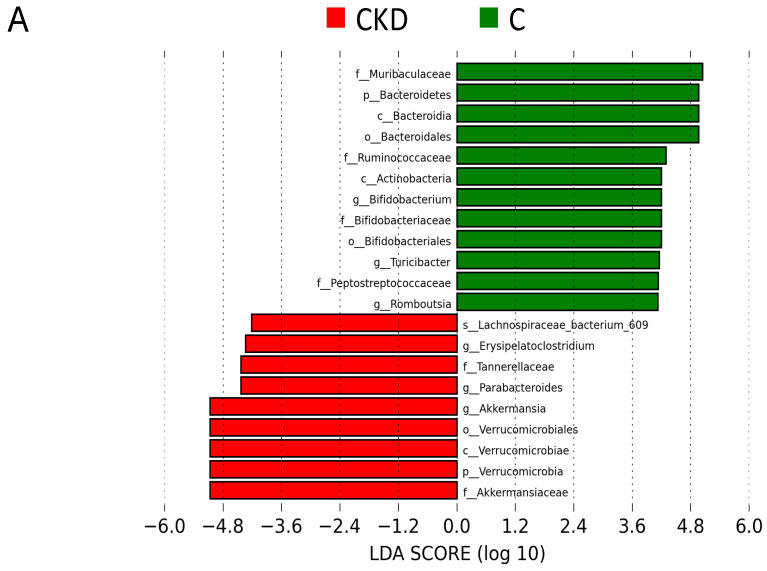

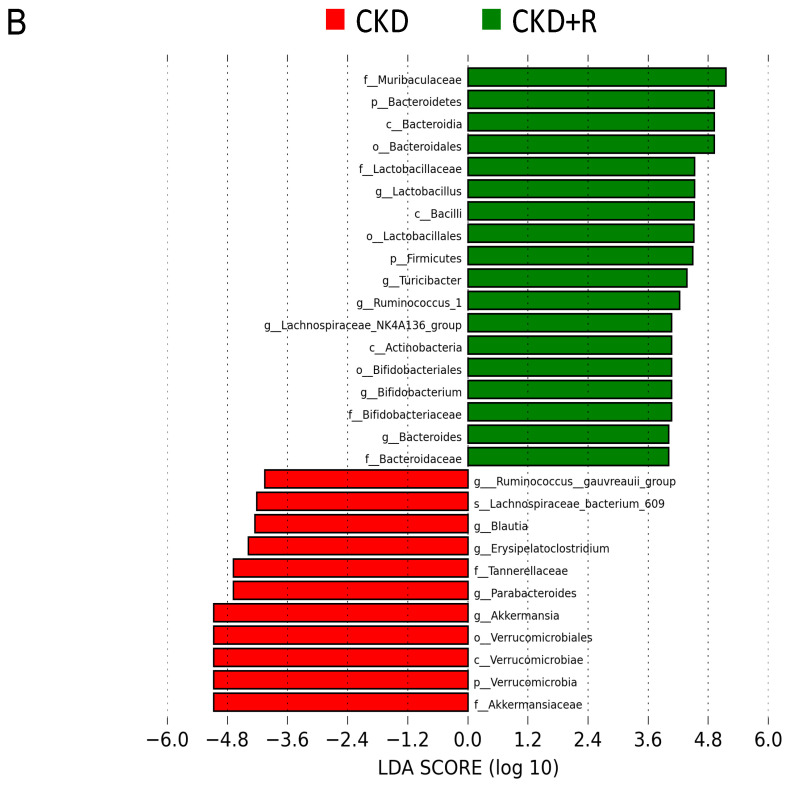
Linear discriminant analysis effect size (LEfSe) was applied to identify enriched bacterial species. The threshold of the linear discriminant was set to 4. Different taxonomic levels of bacteria are given, reaching from phylum (p) and class (c) via order (o) and family (f) down to genus (g) and species (s). The most enriched and depleted bacterial taxa in (**A**) the CKD (red) versus the C group (green) and (**B**) the CKD (red) versus the CKD+R group (green) are shown.

Furthermore, we applied the linear discriminant analysis effect size (LEfSe) algorithm for microbial marker detection (Figure 5). The LEfSe analysis identified a higher abundance of the genus *Akkermansia*, whereas a lower abundance of the genus *Bifidobacterium* in the CKD group vs. the controls (Figure 5A). We observed that resveratrol significantly increased the abundance of the genera *Lactobacillus* and *Bifidobacterium* in the CKD+R group compared with the CKD group (Figure 5B).

### 3.3. TMA, TMAO, and DMA Levels in the Plasma

**Figure 6:**
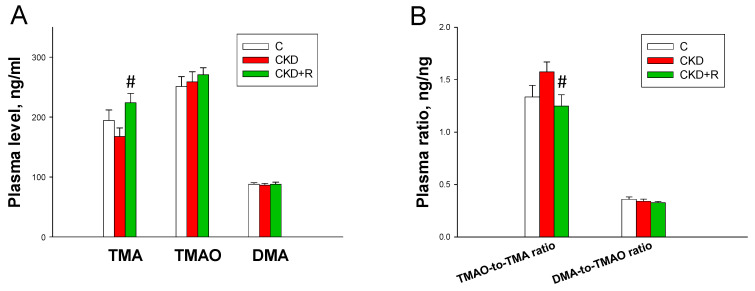
Effects of maternal CKD and R treatment on the trimethylamine (TMA)-trimethylamine-*N*-oxide (TMAO) pathway in male offspring at 12 weeks of age. (**A**) Plasma levels of TMA, TMAO, and dimethylamine (DMA). (**B**) The TMAO-to-TMA and DMA-to-TMAO ratios in the plasma. *N* = 7–8/group; # *p* < 0.05 vs. CKD.

To determine whether the protective effects of resveratrol coincided with alterations of microbiota-derived metabolites, the TMA-TMAO pathway was examined (Figure 6). As a result, TMA, TMAO, and DMA levels in the plasma were comparable between the CKD and C groups (Figure 6A). Resveratrol significantly increased plasma TMA levels in the CKD+R group compared with the CKD group. There was a trend towards a lower plasma TMAO-to-TMA ratio, a measure of FMO activity, in this group (Figure 6B). However, the DMA-to-TMAO ratio (representing TMAO-metabolizing activity) was comparable among the three groups. Taking into account that the plasma TMA level was increased but the TMAO-to-TMA ratio was decreased in the CKD+R group, our data suggest that resveratrol treatment might increase gut bacterial production or decrease TMA-to-TMAO conversion. 

### 3.4. SCFAs and Receptors

**Figure 7:**
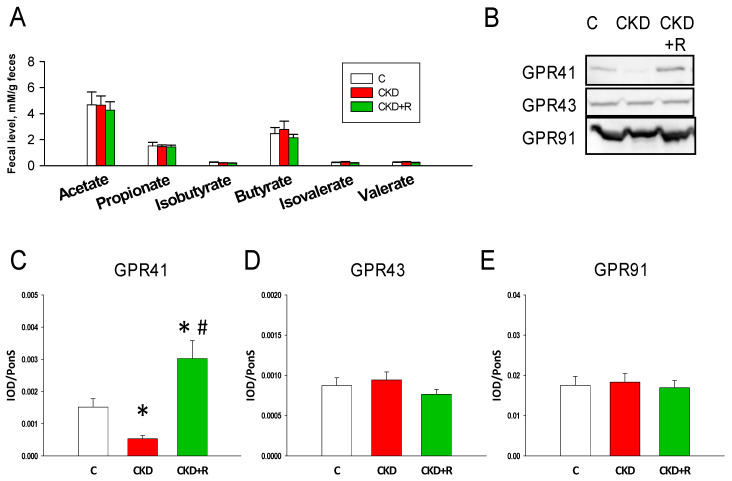
(**A**) Effects of maternal chronic kidney disease (CKD) and resveratrol (R) treatment on fecal levels of short chain fatty acids (SCFAs) in male offspring at 12 weeks of age. (**B**) Representative Western blots and relative abundance of (**C**) GPR41 (45 kDa), (**D**) GPR43 (47 kDa), and (**E**) GPR91 (38 kDa) in male offspring’s kidneys at 12 weeks of age. *N* = 7–8/group; * *p* < 0.05 vs. C; # *p* < 0.05 vs. CKD.

We further investigated whether maternal CKD and resveratrol affect fecal SCFA levels and SCFA receptors in the offspring’s kidneys (Figure 7). As shown in Figure 7A, fecal levels of acetate, propionate, isobutyrate, butyrate, isovalerate, and valerate were not different among the three groups. However, renal GPR41 levels were lower in the CKD group than in the controls, which perinatal resveratrol therapy prevented (Figure 7C). GPR43 and GPR91 abundance were comparable among the three groups (Figure 7D,E).

### 3.5. NO Pathway and Oxidative Stress

As shown in Table 2, plasma L-citrulline levels were higher in the CKD+R group vs. the controls. CKD caused a decrease in L-arginine levels in the CKD group, which was restored by resveratrol treatment. Plasma ADMA and SDMA levels were not different among the three groups. The L-arginine-to-ADMA ratio was higher in the CKD+R group compared with the CKD group.

**Figure 8:**
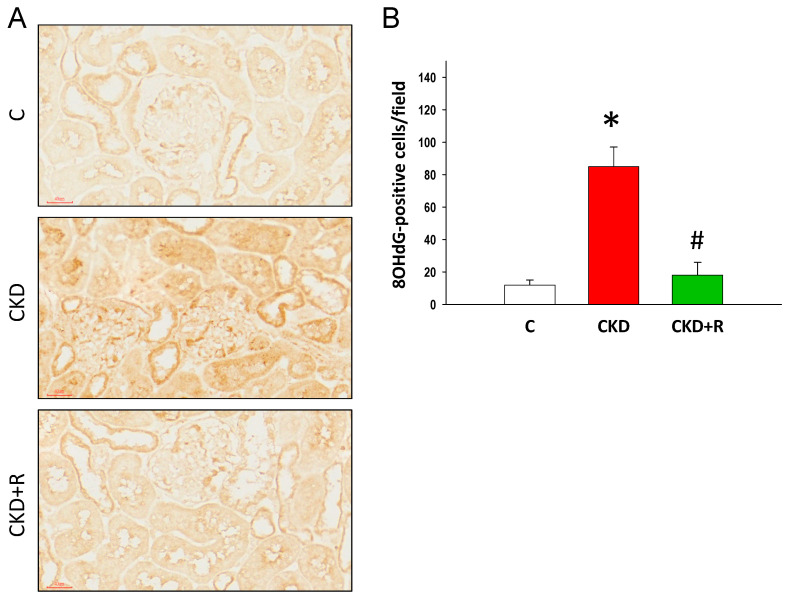
(**A**) Representative light micrographs illustrating 8-hydroxy-2′-deoxyguanosine (8-OHdG) expression in the offspring’s kidneys; Bar = 40 μm. (**B**) Quantitative analysis of 8-OHdG-positive cells per microscopic field; * *p* < 0.05 vs. C; # *p* < 0.05 vs. CKD.

We immunohistochemically stained for 8-OHdG, a marker of oxidative DNA damage, in the offspring’s kidneys (Figure 8A). 8-OHdG was strongly positive in the CKD group vs. controls (Figure 8B). The increased levels of renal 8-OHdG expression were restored by resveratrol treatment. 

## 4. Discussion

In the current study, we evaluated the protective roles of perinatal resveratrol therapy in hypertension programmed by maternal CKD through mediation of the gut microbiota, microbial metabolites, and the NO pathway. Our key findings are as follows: (1) perinatal resveratrol therapy protected against hypertension programmed by maternal adenine-induced CKD in adult male offspring; (2) maternal CKD induced hypertension in adult offspring was associated with alterations in the gut microbiome, like decreased α-diversity and an increased F/B ratio; (3) CKD and resveratrol differentially shaped the gut microbiota profile, leading to three distinct enterotypes; (4) resveratrol therapy increased plasma TMA levels but decreased the plasma TMAO-to-TMA ratio; (5) the protective effects of resveratrol were related to the increases in plasma L-arginine levels and the L-arginine-to-ADMA ratio (AAR), and the decrease of oxidative stress; (6) resveratrol therapy protected adult offspring against hypertension, which coincided with increased genera *Lactobacillus*, *Bacteroides*, *Bifidobacterium*, *Roseburia*, and *Ruminococcaceae* but decreased genus *Akkermansia*; and (7) the LEfSe analysis revealed that *Lactobacillus*, *Bifidobacterium*, and *Ruminococcaceae* could be marker genera.

To our knowledge, no prior research has examined the protective effects of perinatal resveratrol therapy on offspring hypertension arising from maternal CKD. Our results go beyond previous studies demonstrating the anti-hypertensive effects of resveratrol on established hypertension [22,23]; we first shifted resveratrol treatment from adulthood to the perinatal period to protect adult offspring against maternal CKD-induced programmed hypertension. Of note, the antihypertensive effect of resveratrol manifested from 6 weeks of age onwards (i.e., 3 weeks after cessation of treatment). These findings indicate that any reduction in the SBP after perinatal resveratrol therapy is more likely due to reprogramming instead of an acute effect. The results of the present study are in line with those of our previous study showing that maternal adenine-induced CKD leads to the risk of offspring developing hypertension and renal hypertrophy, two common characteristics of early CKD [4,30].

In the present study, the protective effects of perinatal resveratrol therapy against maternal CKD-induced programmed hypertension are related to an altered gut microbiome and microbe-derived metabolites. Previous investigations have reported that resveratrol could modulate the gut microbiota in different ways, such as increasing the abundance of beneficial probiotics [32], reducing the F/B ratio [33], reducing TMAO levels by inhibiting the growth of TMAO-producing bacteria [32], and increasing the production of SCFAs [34]. Our study provides further evidence for an association between resveratrol’s reprogramming effect and gut microbiome remodeling, including an increased abundance of beneficial microbes, mediation of the TMA-TMAO pathway, and alterations of the SCFA receptors. 

First, maternal CKD reduced microbial richness and increased the F/B ratio, which were restored by perinatal resveratrol therapy. Since decreases in the microbial richness and increases in the F/B ratio have been shown in animal models of hypertension [35], perinatal use of resveratrol might benefit programmed hypertension due to, at least in part, its long-term influence on reshaping the offspring’s gut microbiome. Second, the results of the present study showed resveratrol increased abundance of genera *Lactobacillus* and *Bifidobacterium*. Species belonging to the genera *Lactobacillus* and *Bifidobacterium* are well-known beneficial probiotic microbes for intestinal health in humans and animals [36]. The findings of this research are consistent with prior reports showing that resveratrol could increase the abundance of *Lactobacillus* and *Bifidobacterium* [32], and that certain probiotic strains like *Lactobacillus* have shown hypotensive effects in developmental programming models of hypertension [18,37]. In line with a previous study showing that hypertensive patients were mainly characterized by increased proportions of *Akkermansia* but decreased relative abundance of *Roseburia* [38], we found that CKD-induced programmed hypertension coincided with increased genus *Akkermansia* but decreased genus *Roseburia*. Third, we observed that perinatal resveratrol therapy increased plasma TMA levels in adult offspring. Resveratrol also decreased TMAO-to-TMA ratio in the CKD+R group. These findings suggested resveratrol’s benefits for programmed hypertension might be associated with the prevention of TMAO accumulation. At the genus level, TMA and/or TMAO levels are negatively correlated with *Akkermansia* and positively correlated with *Ruminococcaceae* [32]. Here, we found that resveratrol increased plasma TMA levels, combined with decreased abundance of the genus *Akkermansia* and increased *Ruminococcaceae*. Overall, these observations indicate that certain bacteria populations involved in TMA metabolism were affected by resveratrol therapy. Fourth, the beneficial effects of perinatal resveratrol therapy also contributed to mediation of the SCFA receptors. According to our data, maternal CKD-induced offspring hypertension coincided with a decrease in renal GPR41 protein levels. Although perinatal resveratrol therapy had negligible effects on fecal SCFAs levels, it upregulated the SCFA receptor GPR41 in the offspring’s kidneys. With regard to the current literature, there is no reported direct interaction between resveratrol and SCFA receptors. Given that GPR41 null mice are hypertensive [39], the beneficial effect of resveratrol against maternal CKD-induced programed hypertension might be related to regulation of GPR41 and the overall balance of vasodilation and vasoconstriction shifts towards vasodilatation.

Another possible beneficial effect of resveratrol therapy may be related to the reduction of oxidative stress. This is in line with previous studies that show adenine-induced CKD is related to induction of oxidative stress [40,41]. Our results showed that maternal CKD induced oxidative DNA damage in the offspring’s kidneys, which is represented by increased 8-OHdG immunostaining. Our results showed that the increased levels of renal 8-OHdG induced by maternal CKD were restored by resveratrol therapy. Moreover, resveratrol’s protection against hypertension programmed by maternal CKD may be related to restoration of the NO pathway. It has been well described in animal models that the NO system plays a decisive role in developmental programming of hypertension and kidney disease [19]. We observed that resveratrol therapy increased plasma levels of L-arginine and the L-arginine-to-ADMA ratio (AAR), a measure of NO bioavailability. Our results are unsurprising, in view of previous studies showing resveratrol’s benefits for hypertension related to restoration of the NO pathway in a variety of developmental hypertension models [25,42]. 

A potential limitation of the current study is the inability to conduct the control+R group. The reason is clinical trials in humans have shown that resveratrol has an excellent safety profile [43]. Nevertheless, the long-term programmed effects of resveratrol on normal controls deserve further elucidation. Additionally, it remains to be determined whether the changes in body weight by resveratrol therapy observed in the current study are beneficial or harmful. Another limitation is that we did not analyze the maternal gut microbiota, as we mainly focused on the offspring’s gut microbiota in this study. Resveratrol and metabolites can enter systemic circulation and be absorbed by peripheral tissues to execute their multi-organ effects [44]. Accordingly, whether perinatal resveratrol therapy may have beneficial effects on CKD-induced hypertension through the altered maternal gut microbiota and metabolites, lactation, or other routes deserve further clarification. Moreover, we only determined the gut microbiome in adult offspring, but not in the offspring at weaning. Additional studies are required to elucidate whether the different stages of development experienced by offspring could influence their gut microbiota, and whether gut microbiota and their metabolites are altered differently by resveratrol at different developmental stages.

## 5. Conclusions

We conclude that perinatal resveratrol therapy protects male adult offspring against hypertension arising from maternal CKD, primarily through mediation of the gut microbiota and their metabolites and the NO pathway. Our results open a new avenue of research regarding the reprogramming effects of resveratrol against hypertension and indicate that the gut microbiota and their metabolites may become a potential target for reprogramming interventions to stop the global epidemic of hypertension and kidney disease.

## Figures and Tables

**Table 1:** Weights, blood pressure, and renal function.

Groups	C	CKD	CKD+R
Body weight (BW) (g)	357 ± 11	384 ± 12	441 ± 17 *#
Left kidney weight (g)	1.54 ± 0.07	1.8 ± 0.01 *	2.14 ± 0.08 *#
Left kidney weight/100 g BW	0.43 ± 0.01	0.47 ± 0.02 *	0.49 ± 0.02 *
Systolic blood pressure (mmHg)	131 ± 1	145 ± 1 *	136 ± 1 #
Diastolic blood pressure (mmHg)	83 ± 2	91 ± 2 *	88 ± 1
Mean arterial pressure (mmHg)	99 ± 2	109 ± 2 *	104 ± 1
Creatinine (μM)	19.2 ± 0.7	22.6 ± 1.5	17.6 ± 0.8

*N* = 7–8/group; * *p* < 0.05 vs. control ©; # *p* < 0.05 vs. CKD.

**Table 2:** Plasma levels of analytes involved in the NO pathway.

Groups	C	CKD	CKD+R
L-Citrulline (μM)	41.3 ± 4	44.6 ± 4.1	55.5 ± 3.1 *
L-Arginine (μM)	173 ± 8.2	148.6 ± 7.5 *	178.84 ± 7.8 #
ADMA (μM)	1.69 ± 0.2	1.54 ± 0.2	1.13 ± 0.26
SDMA (μM)	1.32 ± 0.2	1.16 ± 0.23	0.89 ± 0.2
L-Arginine-to-ADMA ratio (μM/μM)	114 ± 17	108 ± 17	222 ± 56 #

*N* = 7–8/group; * *p* < 0.05 vs. C; # *p* < 0.05 vs. CKD.
